# Major Latex Protein MdMLP423 Negatively Regulates Defense against Fungal Infections in Apple

**DOI:** 10.3390/ijms21051879

**Published:** 2020-03-10

**Authors:** Shanshan He, Gaopeng Yuan, Shuxun Bian, Xiaolei Han, Kai Liu, Peihua Cong, Caixia Zhang

**Affiliations:** 1 Research Institute of Pomology, Chinese Academy of Agricultural Sciences, Xingcheng 125100, China; hess920309@163.com (S.H.); yuangp329@163.com (G.Y.); bb18236767941@163.com (S.B.); hanxiaolei@caas.cn (X.H.); 15615781850@163.com (K.L.); congph@163.com (P.C.); 2 Key Laboratory of Biology and Genetic Improvement of Horticultural Crops, Xingcheng 125100, China

**Keywords:** major latex proteins, fungal infections, apple (*Malus × domestica*), defense and stress responses

## Abstract

Major latex proteins (MLPs) play critical roles in plants defense and stress responses. However, the roles of *MLPs* from apple (*Malus × domestica*) have not been clearly identified. In this study, we focused on the biological role of *MdMLP423*, which had been previously characterized as a potential pathogenesis-related gene. Phylogenetic analysis and conserved domain analysis indicated that *MdMLP423* is a protein with a ‘Gly-rich loop’ (GXGGXG) domain belonging to the Bet v_1 subfamily. Gene expression profiles showed that *MdMLP423* is mainly expressed in flowers. In addition, the expression of *MdMLP423* was significantly inhibited by *Botryosphaeria berengeriana* f. sp. *piricola* (BB) and *Alternaria alternata* apple pathotype (AAAP) infections. Apple calli overexpressing *MdMLP423* had lower expression of resistance-related genes, and were more sensitive to infection with BB and AAAP compared with non-transgenic calli. RNA-seq analysis of *MdMLP423*-overexpressing calli and non-transgenic calli indicated that *MdMLP423* regulated the expression of a number of differentially expressed genes (DEGs) and transcription factors, including genes involved in phytohormone signaling pathways, cell wall reinforcement, and genes encoding the defense-related proteins, AP2-EREBP, WRKY, MYB, NAC, Zinc finger protein, and ABI3. Taken together, our results demonstrate that *MdMLP423* negatively regulates apple resistance to BB and AAAP infections by inhibiting the expression of defense- and stress-related genes and transcription factors.

## 1. Introduction

Major latex protein (MLP) is a plant-specific protein that was first identified in opium poppy latex and is widely found in other plants [1,2]. The proteins encoded by genes that are homologous to *MLP* are known as MLP-like proteins, and had been identified and isolated from cucumber [3], peach [4], ginseng [5], *Arabidopsis thaliana* [6], melon [7], soybean [8], and grape [9]. The numbers of MLP-like proteins vary between species, for example, *A. thaliana* has 24 MLP-like proteins but grape only has 14 [9].

The MLP protein contains a Bet v_1 structure and the MLP-like proteins make up the second largest family in the Bet v_1 protein superfamily [10]. The members of this superfamily respond to biotic and abiotic stresses and play important roles in plant growth and development, including disease resistance, stress tolerance, and development [11,12,13]. Bet v_1 proteins have a ‘Gly-rich loop′ (GXGGXG) domain and three-dimensional structure similar to the pathogenesis-related protein PR 10. The Gly-rich loop is a broad class of domains with nuclease activity that are widely present in phosphorylated and nucleic acid-binding proteins [14,15]. At present, the biological role of the MLP protein is not clear, but its responses to the expression patterns of biotic and abiotic stresses indicate that it may play important roles in defense or stress responses [16].

When facing on exogenous biological stress, plants rely on innate immune mechanisms. Previous studies have shown that invading pathogens induce the expression of MLP family proteins that help defend the plant primarily using innate immunity and acquired resistance signals [17]. Chen and Dai [18] cloned the cotton *GhMLP* and found that it was rapidly induced by fungal pathogen *Verticillium dahliae*. The expression of *GhMLP423* increased in cotton plants during infection by *Bemisia tabaci*. Using RNA interference to decrease *GhMLP423* expression in cotton during *B. tabaci* infections led to lower peroxidase, and higher superoxide dismutase activity, respectively [19]. Additionally, another study reported that the expression of *MdMLP423* (gi|658009573) in apples was down-regulated by infection with the *A. alternata* apple pathotype (AAAP), a fungal pathogen that causes *Alternaria* blotch disease [20]. Subsequently, Zhang et al. [21] used differential proteomics analysis to study apple varieties with different levels of resistance to the *Alternaria* blotch disease, and found that the expression of *MdMLP423* was down-regulated after infection by AAAP. Similar studies in tobacco revealed that the expression of *NbMLP423*—the tobacco homolog of *MdMLP423*—was significantly up-regulated after infection by *A. alternata* [22] Furthermore, overexpressing of NbMLP423 in tobacco significantly enhanced the plants resistance to *A. alternata* and promoted the up-regulation of PR10 (NtPR10).

Plant hormones play various roles in all stages of plant growth and development. Studies have shown that salicylic acid (SA), jasmonic acid (JA), abscisic acid (ABA), gibberellin (GA), ethylene (ETH), and other plant hormones play crucial roles in the defense signaling pathway, and interact with other signaling pathways [23,24,25]. Previous studies have reported that exogenous SA significantly reduced the expression of the *MLP151* gene [5], and exogenous SA increased the transcription of *MLP* genes in grapes [9], which demonstrate that MLP proteins respond to salicylic acid and are important in plant resistance to biotic stress. ABA plays crucial roles in regulating plant development [26]. Expression of *AtMLP43* in *A. thaliana* was down-regulated by exogenous ABA, and transgenic plants overexpressing *AtMLP43* had lower germination rates compared with wildtype plants [27]. Ethylene affects many processes of plant growth and development and plays important roles in resisting a series of biotic and abiotic stresses [20,28,29]. By identifying the *PpMLP1* gene in peach, Ruperti et al. [4] found that ethylene can down-regulate the expression of *PpMLP1*.

Although MLP proteins are involved in various stress responses in plants, the molecular mechanism of the *MdMLP423* (gi|658009573) response to biotic stress is not clear. In this study, we cloned the *MdMLP423* gene and identified its molecular role. We used RNA-sequencing (RNA-seq) to further analyze the transcriptome of transgenic apple tissue overexpressing *MdMLP423*, which increased our understanding of the changes in the expression of genes that are closely related to *MdMLP423* activity, as well as the possible causes for sensitivity to *Botryosphaeria berengeriana* f. sp. *piricola* (BB) and AAAP infections. These two pathogens are major fungal diseases in China, usually leading to enormous damage to apple production [30]. Previous studies have shown that the ‘Golden Delicious’ (GD) apple cultivar is a more susceptible to BB than the ‘Huayue’ (HY) cultivar [31,32], and it is also more susceptible to AAAP than ‘Hanfu’ (HF) [33,34]. ‘GD’, ‘HY’, and ‘HF’ are important cultivars in China and this study has important significance for further resistance breeding.

## 2. Results

### 2.1. Isolation and Sequence Analysis of the MdMLP423 Gene

**Figure 1:**
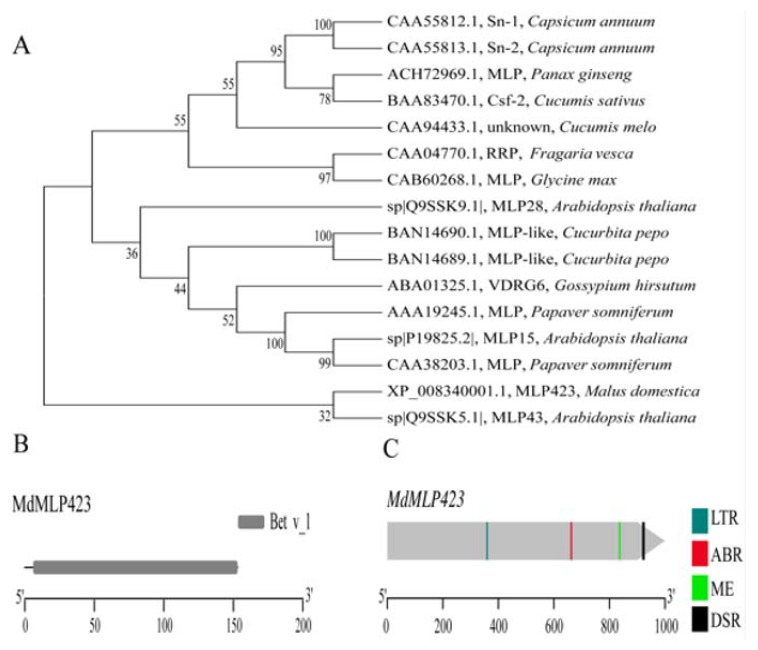
Phylogenetic analysis, protein structure and *cis*-acting elements of MdMLP423. (**A**) Phylogenetic analysis of the MdMLP423 protein with its orthologous genes in other plant species. (**B**) Conserved domain analysis of the MdMLP423 protein. (**C**) Various *cis*-acting elements of the MdMLP423 promoter.

In a previous study we analyzed the iTRAQ-based proteomics profiles of ‘Huayue’ apple leaves in response to AAAP infection, and we found that MdMLP423 is a potential allergy related protein, that is involved in stress responses in fruit trees [21]. Here, we cloned the *MdMLP423* gene from the ‘Golden Delicious’ apple variety. The coding sequence of *MdMLP423* was 462 bp in length, and it encoded a putative protein of 153 amino acids with a calculated molecular mass of 16.92 kDa and an isoelectric point of 4.97. SMART analysis showed that MdMLP423 contained a Bet v_1 domain (residues 3–153; Figure 1B).

The phylogenetic analysis showed that the MdMLP423 protein (XP_008340001.1) was closely connected with AtMLP43 (Q9SSK5.1; Figure 1A) from *A. thaliana*, but shared only 35.85% identity with AtMLP43 (Figure S1). It has been previously reported that many MLP proteins have low sequence similarity to each other but still retain a similar three-dimensional structure [10,35].

We analyzed the promoter of *MdMLP423* to investigate its *cis*-acting elements. Several stress-related *cis*-acting elements were identified including one low-temperature responsiveness (LTR) element and one defense and stress responsiveness (DSR) element (Figure 1C). Additionally, *cis*-acting elements related to hormone responses, such as the ABRE involved in ABA response, and the CAT-box related to meristem expression were also detected (Figure 1C). These results indicated that *MdMLP423* might be associated with stress responses as well as plant development.

### 2.2. Expression of MdMLP423

**Figure 2:**
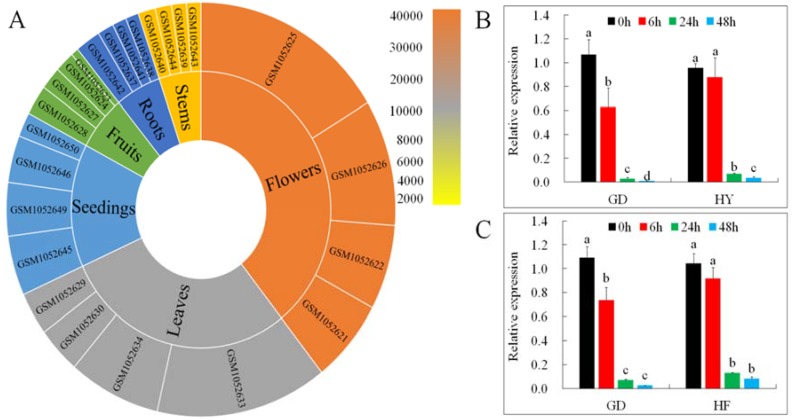
Expression patterns of *MdMLP423*. (**A**) Expression patterns of *MdMLP423* in root, stem, leaf, flowers, fruit, and seedings of *Malus × domestica*. (**B**) qRT-PCR of *MdMLP423* ‘GD’ and ‘HY’ cultivars at 0, 6, 24, and 48 h post-inoculation with *Botryosphaeria berengeriana* f. sp. *piricola* (BB). (**C**) qRT-PCR of *MdMLP423* at 0, 6, 24, and 48 h post-inoculation with *Alternaria alternata* apple pathotype (AAAP). The different lowercase letters indicate significant difference at *p* < 0.05.

Gene expression profiles put forward useful clues to understanding a gene’s role. We examined *MdMLP423* transcripts from different tissues (root, stem, leaf, flower, fruit, and seedling) and under different biotic stresses (including BB and AAAP infections) using the GEO database (https://www.ncbi.nlm.nih.gov/geo/query/acc.cgi?acc=GSE42873) and qRT-PCR. The highest accumulation of *MdMLP423* was found in flowers, followed by leaves and seedings, and lower expression levels were observed in roots and stems (Figure 2A).

*MdMLP423* was markedly down-regulated in response to BB infection in both the ‘GD’ and ‘HY’ cultivars, and at 48 h its expression was nearly 107.9-fold and 25.5-fold lower compared with non-inoculated controls, respectively (Figure 2B). AAAP infection also decreased *MdMLP423* in both cultivars and by 48 h its relative expression was nearly 41.2-fold and 12.6-fold lower in ‘GD’ and ‘HF’, respectively (Figure 2C).

### 2.3. Overexpression of MdMLP423 in Apples

**Figure 3:**
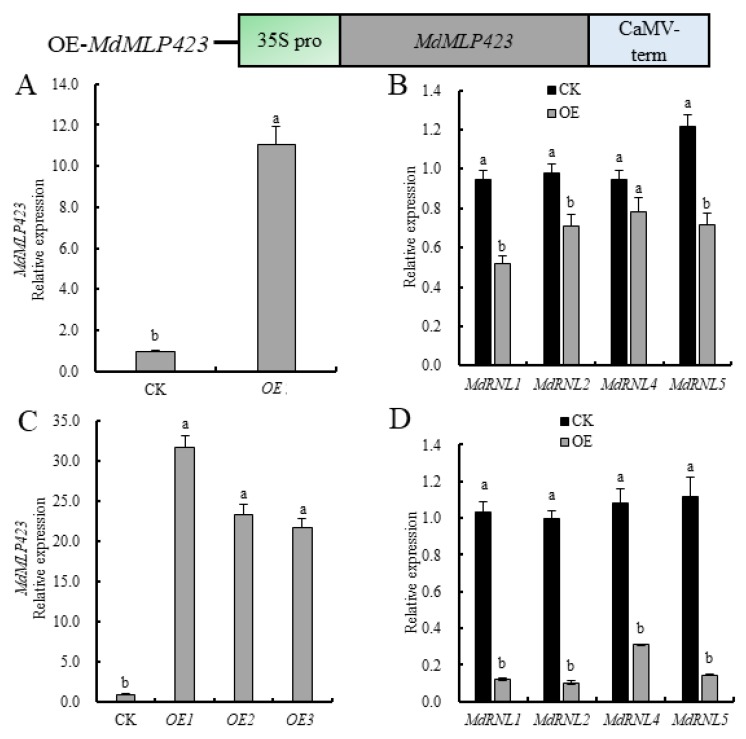
Overexpressing *MdMLP423* in apple decreases the expression of *MdRNLs*. (**A**) Transient overexpression of *MdMLP423* in ‘GD’ leaves. (**B**) Transient expression of *MdRNLs* in ‘GD’ leaves. (**C**) Expression of *MdMLP423* in apple calli from stable genetic transformation. (**D**) Expression of *MdRNLs* in apple calli from stable genetic transformation. The different lowercase letters indicate significant difference at *p* < 0.05.

Since the expression of *MdMLP423* was inhibited under different biotic stresses, we explored whether *MdMLP423* contributed to plant stress resistance. Therefore, we transiently overexpressed *MdMLP423* in ‘GD’ leaves and assessed the expression of four known disease-resistant genes (*MdRNL1*, *MdRNL2*, *MdRNL4*, and *MdRNL5*) [32]. There was a transient but statistically significant 11.0-fold increase in *MdMLP423* expression in leaves compared with the CK (Figure 3A), and all four *MdRNL* genes were down-regulated (1.2–1.8-fold lower; Figure 3B).

To further evaluate the role of *MdMLP423*, we generated transgenic apple calli overexpressing *MdMLP423*. Three independent *MdMLP423* transgenic calli lines (OE-1, OE-2, and OE-3) with high levels of *MdMLP423* expression (21.8–31.8-fold higher than the control (CK) non-transgenic calli) were used for analysis (Figure 3C). The *MdRNLs* were down-regulated (3.5–9.5-fold) in all three transgenic lines (Figure 3D), suggesting that *MdMLP423* might play a negative regulatory role in biotic stresses.

**Figure 4:**
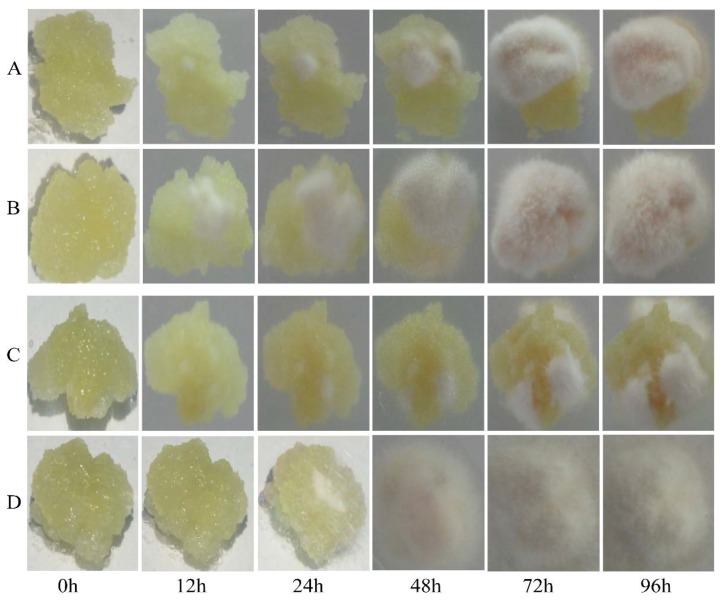
Infection level of calli inoculated with BB and AAAP. Photographs of MdMLP423-overexpressing calli (**A**,**C**) and CK calli (**B**,**D**) at 0, 12, 24, 48, 72, and 96 h post-inoculation with BB (**A**,**B**) or AAAP (**C**,**D**).

Next, we compared the levels of fungal infection in the transgenic and CK calli after inoculation with AAAP or BB. A small amount of fungal mycelia was observed on the surface of transgenic calli infected by BB, but not AAAP, within 12 h post-inoculation (HPI). By 24 HPI mycelium were also observed on the AAAP-inoculated calli and the color around the inoculation sites became darker. At 48 HPI, the surface of the calli inoculated with BB or AAAP were almost completely covered with mycelia and the mycelia had turned yellow. The mycelium had changed color from yellow to khaki by 72 HPI. At 96 HPI the mycelium were brown and extended from the calli to the surface of culture medium (Figure 4B,D). For the CK calli inoculated with AAAP or BB, the pathogenetic time was late and the degree of the disease was mild when compared with the transgenic calli. There was no obvious change within 12 HPI, and a small amount of mycelia was observed at 24 HPI. The color around the inoculation sites became darker at 72 HPI and at 96 HPI almost half of the calli surface was covered with white mycelium (Figure 4A,C).

### 2.4. RNA-Seq Data, DEG Profiles, and Functional Annotation of DEGs

**Figure 5:**
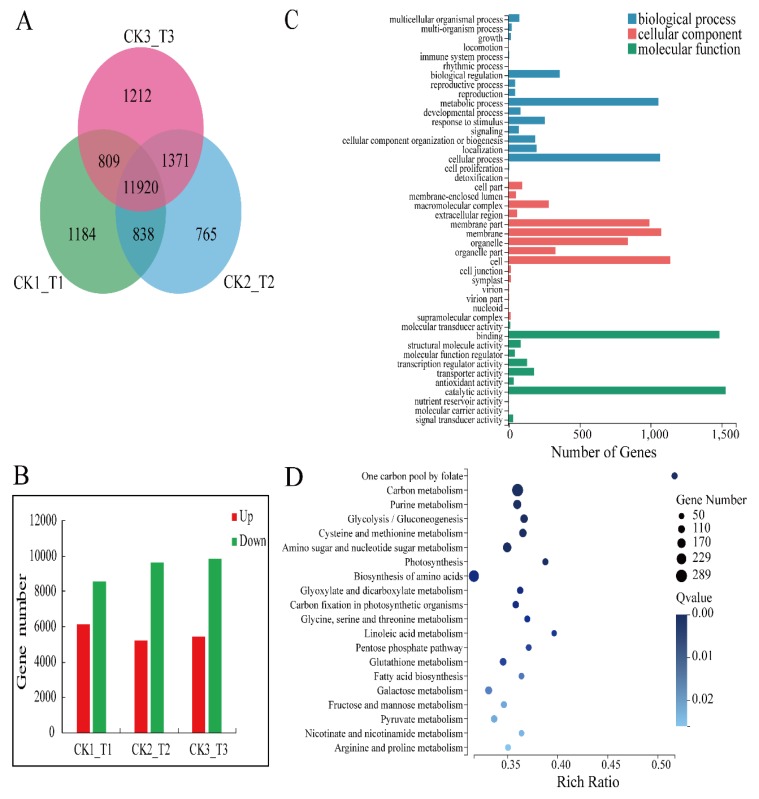
Analysis of differentially expressed genes (DEGs) of transgenic calli and CK. (**A**) Venn diagram of DEGs in comparative groups CK_1/T_1, CK_2/T_2, and CK_3/T_3. (**B**) The statistics of DEGs of comparative groups CK_1/T_1, CK_2/T_2, and CK_3/T_3. (**C**) GO categories analysis of DEGs. (**D**) Top 20 KEGG pathways enrichment analysis of DEGs (according to the Q value).

We used an RNA-seq approach to identify the genes that make the transgenic calli more susceptible to fungal infection. We extracted mRNA from 3 transgenic calli lines and 3 CK lines to construct 6 separate cDNA libraries. The results showed that the mean output data of all samples was 10.22 Gb, and the mean match percentage of all samples to reference genomes and gene sets were 89.94% and 82.46%, respectively (Table S1). In total, 40,113 genes were identified, including 36,753 known genes and 3430 predicted new genes. We screened 11,920 differentially expressed genes (DEGs) in the comparison groups (Figure 5A) and the ratio of down-regulated genes to up-regulated genes was 1.7 (Figure 5B).

GO categories were developed to assess DEG functions. All of the DEGs were classified into 44 functional categories, including biological processes (18), cellular components (15), and molecular functions (11; Figure 5C). Of the genes involved in biological processes, those regulating cellular processes were the most enriched in the transgenic calli. Genes encoding cell components were the most enriched of the cellular component genes. Genes encoding proteins with catalytic function were the most enriched of the molecular function genes.

To further evaluate the differences between the *MdMLP423*-overexpressing and CK calli, KEGG pathway enrichment analysis was carried out for the DEGs. The results indicated that 31 KEGG pathways were significantly enriched (*p* value < 0.05; Table S2). Of these, the most enriched orthology (KO) terms were the carbon metabolism (Ko01200, 289 DEPs) and biosynthesis of amino acids (Ko01230, 254 DEGs) in the top 20 pathways (according to the Q value; Figure 5D). Above all, these results indicate that a series of different molecular changes had occurred in the transgenic calli.

### 2.5. Validation by qRT-PCR

**Figure 6:**
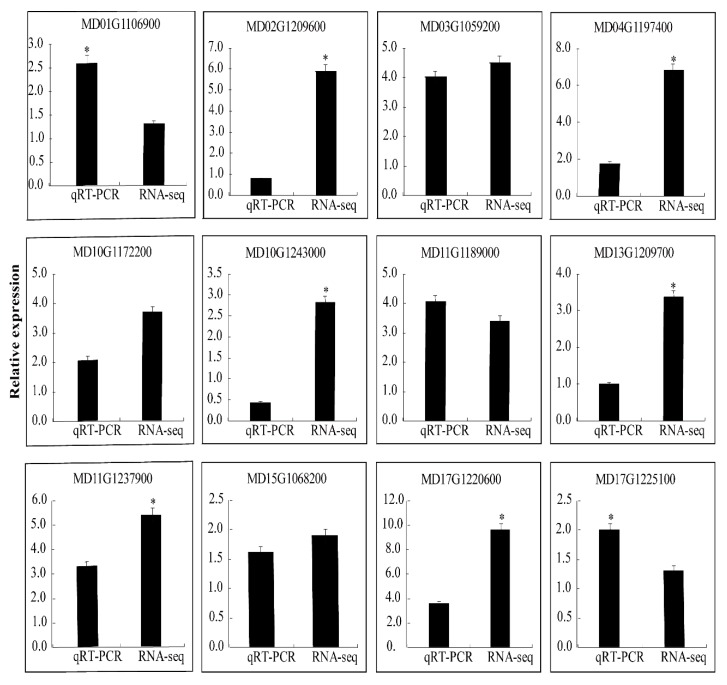
qRT-PCR validation of differential gene expression. The “*” indicate significant difference at *p* < 0.05.

We selected 12 DEGs to evaluate the RNA-Seq data. The results of the qRT-PCR of 12 DEGs were consistent with those of the RNA-Seq, which indicates that there is a high degree of repeatability between the qRT-PCR data and transcriptional abundance (Figure 6). The inconsistencies between the data sets reflect the differences between the two methods.

### 2.6. Changes in DEGs Related to Phytohormones

**Figure 7:**
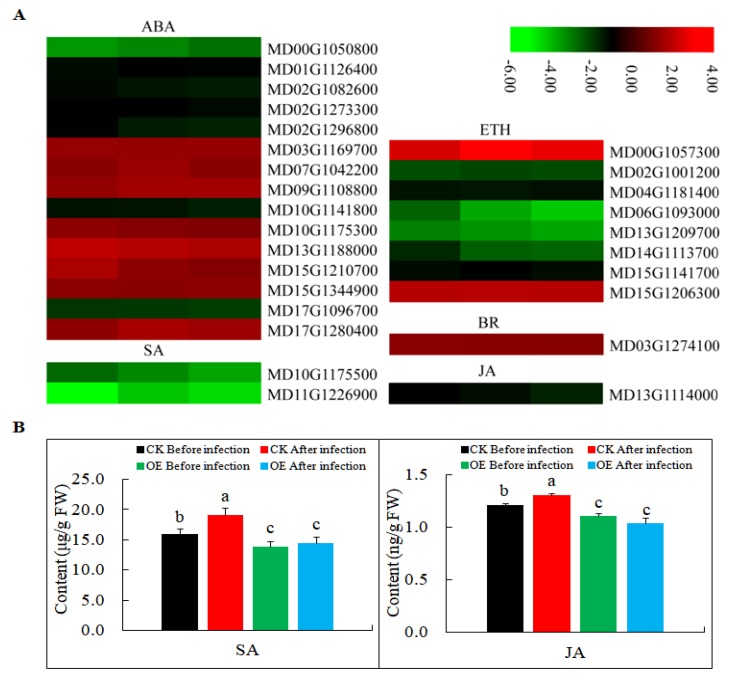
Expression levels of DEGs involved in phytohormone signaling pathways. (**A**) Heatmaps of DEGs involved in phytohormone signaling pathways, including ABA, SA, ETH, BR, and JA signaling pathways in *MdMLP423*-overexpressing calli. The log2|Foldchange| was colored using TBtools (red indicates the DEG was up-regulated in the *MdMLP423*-overexpressing calli compared with the CK calli, green indicates it was down-regulated). Each row represents three independent comparisons (T_1/CK_1, T_2/CK_2, and T_3/CK_3) from left to right. (**B**) The content of SA and JA before and after pathogen inoculation. The different lowercase letters indicate significant difference at *p* < 0.05.

In a previous study, *MdMLP423* (gi|103448744) was found to improve the stress resistance of *A. thaliana* probably through ABA signaling [36]. Therefore, in this study, we first analyzed the changes in the expression of phytohormone-related genes. Most of the genes involved in ETH, and genes involved in SA and JA signaling was down-regulated in *MdMLP423* transgenic calli (Figure 7A), and the contents of SA and JA existed similar results (Figure 7B). ABA plays important regulatory roles in plant–microbe interactions and we found that the DEGs that responded to ABA signal transduction pathways, such as *MD10G1175300* and *MD15G1344900*, were all up-regulated (Figure 7). Additionally, the DEG (*MD03G1274100*) encoding BR signaling—another fundamental regulator of plant immunity—was up-regulated.

### 2.7. Changes in DEGs Related to Cell Wall

**Figure 8:**
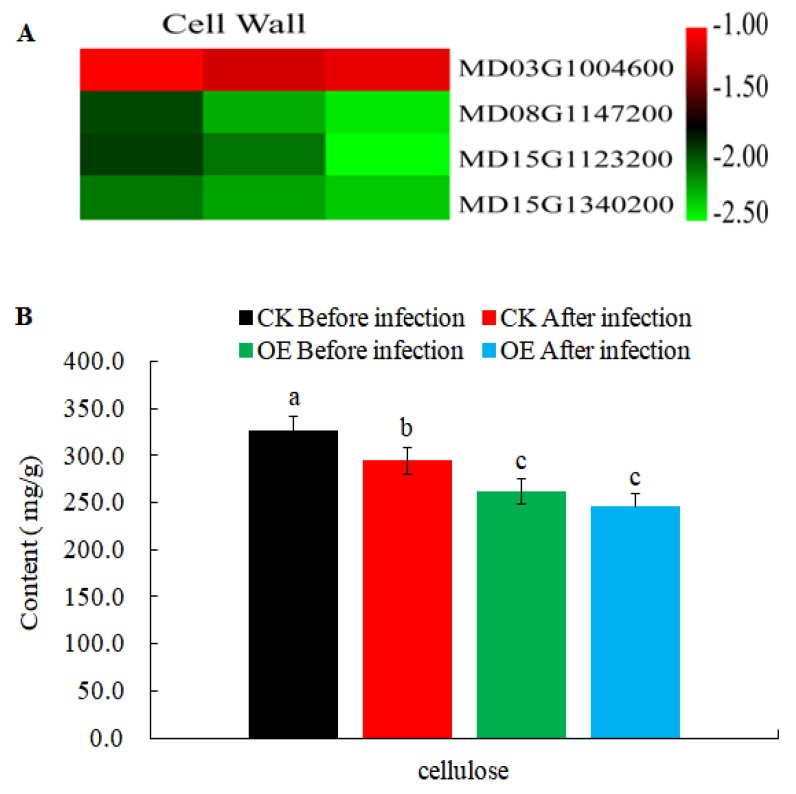
Expression levels of DEGs involved in cell wall reinforcement. (**A**). Heatmaps of DEGs involved in cell wall reinforcement. The log2|Foldchange| was colored using TBtools (red indicates the DEG was up-regulated in the *MdMLP423*-overexpressing calli compared with Table S1. CK_1, T_2/CK_2, and T_3/CK_3) from left to right. (**B**). The content of cellulose before and after pathogen inoculation. The different lowercase letters indicate significant difference at *p* < 0.05.

In order to further explore the changes of resistance-related genes in transgenic calli, some resistance-related DEGs (Table S3) were obtained from 11,920 DEGs crossed with the PRG database (Plant Resistance Gene Database, PRGdb, http://prgdb.crg.eu/). Plant cell walls act as a physical barrier to protect cells from pathogens, and biosynthesis of cellulose (the major component of cell walls) to make primary and secondary cell walls involves different cellulose synthases. Our results revealed that the homologs of cellulose synthase A (*CESA*) genes in apple (*MD08G1147200*, *MD15G1123200*, and *MD15G1340200*) were all down-regulated and the content of cellulose reduced during AAAP and BB infections (Figure 8). This suggested that the biosynthesis of cellulose was disturbed in transgenic calli and this may contribute to the *MdMLP423*-overexpressing lines being more susceptible to these infections.

### 2.8. Changes in DEGs Related to Transcriptional Factors

**Figure 9:**
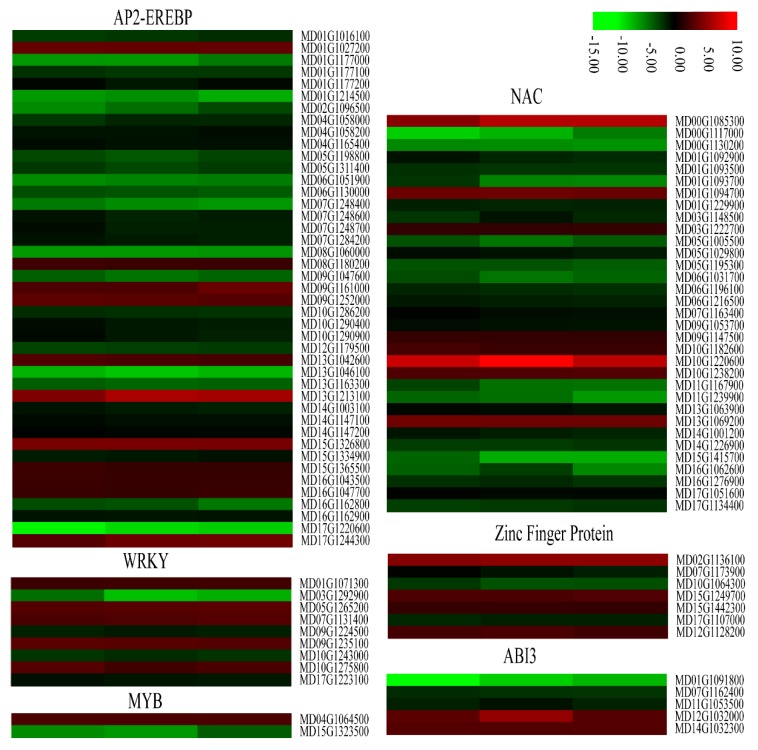
Heatmaps of DEGs encoding transcriptional factors. The log2|Foldchange| was colored using TBtools (red indicates the DEG was up-regulated in the *MdMLP423*-overexpressing calli compared with the CK calli, green indicates it was down-regulated). Each row represents three independent comparisons (T_1/CK_1, T_2/CK_2, and T_3/CK_3) from left to right.

Some plant transcription factors including AP2-EREBP, WRKY, MYB, NAC, Zinc finger proteins, and ABI3 play important regulatory roles in defense against stress. We found that most *AP2-EREBPs*, *NACs*, and some genes encoding *WRKYs*, *MYBs*, *Zinc finger proteins*, and *ABI3s* were significantly down-regulated in the calli overexpressing *MdMLP423* compared with the CK calli (Figure 9). For example, 32 of the 58 AP2-EREBP genes in our transcriptome data (Table S4), and 25 of the 48 NAC family-members, were significantly down-regulated in the transgenic calli. Taken together, these results indicated that overexpressing *MdMLP423* might change the expression of genes that encode resistance-related transcription factors, resulting in increased susceptibility to fungal infections.

### 2.9. Changes in DEGs Related to Defense-Related Proteins

**Figure 10:**
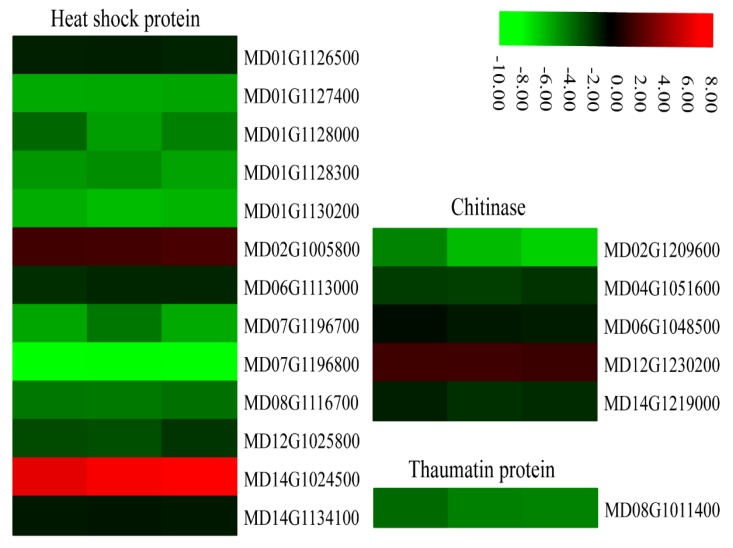
Heatmaps of DEGs encoding defense-related proteins. The log2|Foldchange| was colored using TBtools (red indicates the DEG was up-regulated in the *MdMLP423*-overexpressing calli compared with the CK calli, green indicates it was down-regulated). Each row represents three independent comparisons (T_1/CK_1, T_2/CK_2, and T_3/CK_3) from left to right.

Heat shock proteins (HSPs) are a subfamily of molecular chaperones that are rapidly induced under stress responses. All 13 *HSP* genes identified in our data belonged to the *HSP70* group and 11 of these genes were down-regulated in *MdMLP423*-overexpressing calli compared with the CK calli (Figure 10).

Pathogenesis-related (PR) proteins rapidly accumulate during biotic or abiotic stress. We found that PR thaumatin-like proteins were involved in defense responses regulated by SA, and these proteins had expression patterns that were similar to that of the SA receptor gene (*MD08G1011400*; Figure 7). In addition, several DEGs (*MD02G1209600*, *MD04G1051600*, *MD06G1048500*, and *MD14G1219000*) encoding defense-related chitinases were regulated by JA and showed similar expression patterns to other JA-related genes. Most *PR* genes were down-regulated in the *MdMLP423*-overexpressing calli, which may explain why the transgenic calli are more susceptible to fungal infection.

## 3. Discussion

### 3.1. The Function of MdMLP423

The major latex proteins/ripening-related proteins (MLP/RRPs) subfamily plays important roles in defense and stress responses. In this study we identified the DSR (defense and stress responsiveness) *cis*-acting element of the *MdMLP423* promoter, which may respond to defense- and stress-induced expression of *MdMLP423*. Meanwhile, phylogenetic analysis revealed that MdMLP423 was closely related to AtMLP43, implying that they may have similar functions. For instance, in *A. thaliana*, *AtMLP43* is involved in ABA signaling transduction and directly interacts with SnRK2s by combining its upstream [27]. We identified the ABA-related *cis*-acting element ABR in the *MdMLP423* promoter, suggesting that *MdMLP423* may play roles in the ABA-mediated stress tolerance pathway. Moreover, we found that the MdMLP423 protein contains a Bet v_1 domain, which is closely related to plant response to biotic or abiotic stress and plays crucial roles in plant growth and development, including in disease and stress resistance [10]. These results suggested that *MdMLP423* is a member of the Bet v_1 protein superfamily that may be involved in biotic stress responses.

Many studies have reported that MLP proteins can respond to infection by pathogens. In *A. thaliana*, *AtMLP3*, and *AtMLP28* were induced during *Alternaria* and *Plasmodiophora brassicae* infections [37,38]. In melon, MLP protein accumulated in phloem sap during CMV (cucumber mosaic virus) infection [39]. It also had been reported that *MLPs* were induced by *V. dahliae* in Cotton [18,40,41,42]. The expression of *NbMLP423* in tobacco was significantly up-regulated after infection by tobacco brown spot (*A. alternate*) [22]. However, we found that *MdMLP423* was significantly down-regulated under BB and AAAP infections, suggesting that the roles of MLP proteins may differ between species. Meanwhile, the transient expression and stable overexpression of *MdMLP423* in calli down-regulated the expression of *MdRNLs*, and led to higher levels of BB and AAAP infection in the transgenic calli. These results suggested that *MdMLP423* might negatively regulate plant responses to BB and AAAP infection.

### 3.2. Changes in Resistance-Related Genes

The cell wall is an important barrier against pathogen infection in plants [20,33,43], and has been shown to increase the mechanical strength of the cell wall and resistance to pathogens [44]. Cellulose is the basic component of the cell wall and accounts for 30% of the material in the cell wall of a typical dicot. In this study we found that all the genes related to cellulose synthases were significantly down-regulated in *MdMLP423*-overexpressing plants and we suggest that the weakness of cell wall might be part of the reasons why the transgenic calli were more susceptible to BB and AAAP infection compared with the CK calli.

Phytohormones, such as ABA, SA, ETH, BR, and JA, play important roles in regulating the plant defense signaling network [29]. In *A. thaliana*, ABA has been repeatedly proved to be mainly involved in the negative regulation of plant defenses against paralysis immunity through the antagonistic SA pathway [45,46]. The changes in the expression of genes in ABA signal transduction pathways we observed in this study were consistent with the earlier findings. However, other studies found that ABA mainly acts as a positive regulator of immunity in soybean by stimulating callose deposition [47,48]. We found that some genes involved in ABA signaling were actually down-regulated, such as *MD00G1050800*, *MD01G1126400*, and *MD02G1082600*, which suggests that ABA might play different roles in plant immune responses in different species. It is generally believed that SA mediates defense responses to biotrophic and hemibiotrophic pathogens, whereas, JA and ETH are related to the defense responses to necrotrophs [49,50,51]. A previous study had demonstrated that genes involved in ETH and SA signaling were induced when the apple was challenged by AAAP [20]. However, we found the opposite results and infection levels were higher in *MdMLP423*-overexpressing calli after BB and AAAP infections. Nevertheless, the results do suggest that SA may play important roles in local immune responses against BB and AAAP. Taken together, these results revealed that activities of multiple phytohormone signaling pathways had changed in the transgenic calli and that these changes were intertwined. Thus, the susceptibility of transgenic calli to BB and AAAP infections may be caused by several different pathways.

We found that most of the genes encoding WRKY transcription factors were down-regulated in *MdMLP423*-overexpressing calli. Of these genes, *MD03G1292900*, which has high sequence similarity with *PtrWRKY35*, had the largest fold-change in expression (almost 128-fold) compared with the CK calli. A previous study had reported that *PtrWRKY35* positively regulates JA-mediated defense signaling responses to *Botrytis cinereal* in transgenic *A. thaliana* plants. In our study, we found that the homolog of *PtrWRKY35* in apples may play crucial roles in responses to pathogen infection. *NAC* genes positively regulate basic disease resistance in plants by activating *PR* genes, or negatively regulate plant defense responses by inhibiting the expression of *PR1* [52,53,54,55]. One gene encoding a NAC transcription factor, *MD00G1117000*, was similar to *AtNAC017* and was down-regulated more than 190-fold in transgenic calli compared with CK calli. At the same time a gene that encodes a thaumatin-like proteins encoding gene *MD08G1011400* (*PR5*), was also significantly down-regulated by over 18-fold. In *A. thaliana*, plants with mutated *nac017* were more stress sensitive, suggesting that the lack of NAC transcription factors are not conducive to stress responses [56].

The expression levels of DEGs encoding defense-related proteins including HSPs and chitinases were significantly different in the transgenic calli compared to the CK calli. A previous study reported that the expression of the *TaHSP70* gene in wheat was induced by *Puccinia striiformis*, and it was speculated that *TaHSP70* may participate in the basic defenses of wheat to infection by *P. striiformis* through the JA signal pathway [57]. The expression of the HSP70 proteins is another important factor of apple disease resistance. Several *HSP70* genes were down-regulated in the transgenic calli, suggesting that the *HSP70*-mediated defense signaling pathway may be damaged, resulting in increased susceptibility to fungal infections. When tobacco was inoculated with *A. alternata*, the content of chitinase increased significantly in disease-resistant varieties compared with susceptible varieties. This suggested that chitinase activity might promote resistance to *A. alternata* and other pathogens. Similarly, the genes encoding chitinase and β-glucosidases were down-regulated in our results, suggesting these genes may weaken the defense responses of *MdMLP423*-overexpressing calli.

## 4. Materials and Methods

### 4.1. Plant Materials and Treatment

The leaves came from four-year-old ‘GD’, ‘HF’, and ‘HY’ plants, which were grown in the Institute of Pomology, Chinese Academy of Agricultural Sciences (Xingcheng, China (40°37′ N, 120°44′ E)). The AAAP and BB came from Plant Protection Center of Institute of Pomology, Chinese Academy of Agricultural Sciences. We collected 20-day-old leaves from the three cultivars and used the suspension drop method to inoculate the leaves with the fungi [33]. For each experiment, the leaves were inoculated with liquid containing AAAP or BB in four locations on both sides of the midvein on the back of leaves. Leaves inoculated with sterile water were used as a control. Four groups of leaves were inoculated with fungus liquid or sterile water at the same time and then incubated at 25 °C under a 16 h/8 h light/dark photoperiod in a sterilized glass culture dish. We collected the leaves at 0, 6, 24, and 48 h post-inoculation (HPI) and these the four time points represented the four infectious stages.

All samples were frozen directly in liquid nitrogen and stored at −80 °C before RNA extraction. Each group contained three biological replicates.

### 4.2. Cloning and Sequence Analysis of MdMLP423

The coding sequence of *MdMLP423* was amplified from the cDNA of ‘GD’ using gene-specific primers (5′-ATGGCTACATTTGATG-3′ and 5′-TTAATTAGCCAGAACATAGG-3′). DNAMAN 6.0 software was used to deduce the amino acid sequence of MdMLP423. We used Clustal W [58] to perform the MLP protein sequences alignments, and using MEGA 7.0 software [59] to construct phylogenetic trees by the NJ (neighbor-joining) method with 1000 bootstrap replicates. ExPASy (http://web.expasy.org/compute_pi) was used to predict the molecular weight (Mw) and isoelectric points (pI) of MdMLP423. SMART (http://smart.embl-heidelberg.de) was used to analyze the conserved domain of the MdMLP423 protein sequence. The upstream sequence (1 kb) of the transcription start site of the *MdMLP423* gene was defined as promoter regions, which was extracted from ‘GD’ genome sequence (GDDH13) [60] by the TBtools software [61]. The *cis*-acting elements of promoter were analyzed by the PlantCARE database (http://bioinformatics.psb.ugent.be/webtools/plantcare/html/).

### 4.3. Agrobacterium-Mediated Transient Expression Assays with pRI101-AN Constructs

*Agrobacterium*-mediated expression was performed using the pRI101-AN vector driven by the 35S promoter. The coding region of *MdMLP423* was cloned into the pRI101-AN vector at KpnI/SacI to generate the recombinant vector pRI101-AN:MdMLP423, using the following two primers 5′-GGTACCATGGCTACATTTGATG-3′ and 5′-GAGCTCTTAATTAGCCAGAACATAGG-3′. Then the pRI101-AN vector and the recombinant vector were transformed into the *A. tumefaciens* strain GV3101. The pRI101-AN construct was used in the experiment as a control, and the pRI101-AN:MdMLP423 construct was used as the test construct. According to previous protocol [33], following the addition of 50 μL of 100 μM acetosyringone, the vector-transformed *Agrobacterium* in 50 mL YEP was cultured at 28 °C with shaking at 180 rpm for 5–6 h until OD_600_ = 0.4–0.6. The *Agrobacterium* was then suspended in liquid medium (10 mM MES-KOH [pH 5.2], 10 mM MgCl_2_, 100 μM acetosyringone) and incubated for 3 h before use.

For the transient expression of MdMLp423, 5 20-day-old leaves of ‘GD’ apple were infiltrated in the resuspended bacteria and vacuumed (10 Pa) for 30 min [62], then the leaves were washed 3 times with sterile water and placed in a petri dish covered with filter paper and cultured at 24 °C with a 16 h/8 h light/dark photoperiod. Four days after *Agrobacterium* infiltration, the leaves were sampled and RNA was extracted for gene expression analysis.

For stable expression of *MdMLP423*, the calli of ‘Orin’ apple was first infiltrated in the resuspended bacteria for 15 min [63], and then washed 3 times with sterile water and placed in a petri dish covered with filter paper to absorb the excess water. Then the calli were transferred to the subculture medium (MS + 2.0 mg L^−1^ 2,4-D + 0.5 mg L^−1^ NAA + 2.0 mg L^−1^ 6-BA, pH 5.8) for culture at 22 °C in the dark for 2 days. Next, the calli were transferred to the subculture medium containing 300 mg L^−1^ cephalosporin and 30 mg L^−1^ kanamycin for screening resistant calli. Subsequently, the resistant calli were sub-cultured several times to obtain stable resistant calli. Finally, the calli were sampled, DNA and RNA was extracted and used for PCR identification and gene expression analysis, respectively.

### 4.4. Disease Resistance Identification of Transgenic Materials

In order to identify the resistance of *MdMLP423*-overexpressing transgenic calli, AAAP and BB were used to inoculate calli by the suspension drop method. According to a previously described methods [63], the calli of ‘Orin’ cultivar were used as it is the most mature genetic transformation material and is susceptible to both AAAP and BB [63,64]. The calli were placed in a petri dish with subculture medium and then 20 μL of liquid containing the fungus was placed onto the center of the calli and then the calli were cultured at 24 °C and a 16 h/8 h light/dark photoperiod. Non-transgenic calli were used as a control. Regular images were taken to investigate the time, severity, and speed of the decay and browning the calli, and three biological replicates were performed for each treatment.

### 4.5. RNA Extraction and Sequencing

Total RNA was extracted from the six samples of *MdMLP423*-overexpressing transgenic and CK calli using the RNeasy Plant Mini Kit (Tiangen, Beijing, China) according to the manufacturer’s protocol. For each RNA sample, RIN value ≥ 6.5, A260/A280 ratio ≥ 1.8, and A260/A230 ratio ≥ 1.8. More than 200 pg high-quality RNA was amplified with oligo-dT and dNTPs (KAPA HiFi HS RM, KAPA, MA, USA), then reverse transcribed to cDNA. The average molecule length of PCR product was determined by the Agilent 2100 bioanalyzer instrument (Thermo Fisher Scientific, MA, USA). Then we used a fragment buffer (5× first strand buffer, Invitrogen, CA, USA) to fragment the purified cDNA from the previous step into small pieces by PCR, and the product was purified and selected by the Agencourt AMPure XP-Medium kit (Thermo Fisher Scientific, Massachusetts, USA). An Agilent Technologies 2100 bioanalyzer was used to quantify cDNA. The single strand circle DNA (ssCir DNA) was formatted as the final library. The BGISEQ-500 System (BGI-Shenzhen, China) was used for cDNA libraries sequencing. The read length of the BGISEQ-500 system was 100 bp [65].

The clean reads were obtained by filtering the sequenced data, then the clean reads were mapped to GDDH13_v1.1 database (ftp://ftp.bioinfo.wsu.edu/species/Malus%20%C3%97%20domestica/) with Bowtie2 (http://bowtie-bio.sourceforge.net/Bowtie2/index.shtml). Then, we used the FPKM (fragments per kilobase million) method to the calculate gene expression levels. The raw sequences were deposited in the GEO website (GEO, https://www.ncbi.nlm.nih.gov/geo, accession number: GSE139972). Nr, Swiss-prot, KEGG, and GO databases were used to perform gene annotation and functional assignments. The genes with fold change ≥2.00 and FDR value ≤ 0.001 were defined differentially expressed genes (DEGs). GO enrichment and KEGG pathway enrichment were carried out to identify significantly enriched metabolic pathways and compared with the whole genome background. According to the annotation results and official classification of GO and KEGG, the DEGs were functionally classified, respectively. Generally, it was considered that the function of FDR ≤ 0.01 was significant enrichment. For the transcription factors, we used getorf to detect the ORF of each gene, and then used HMM search to compare ORF with transcription factor protein domain, and then the ability of unigene was identified according to the characteristics of transcription factor family described by PlantTFDB (http://planttfdb.cbi.pku.edu.cn).

### 4.6. Quantitative Real Time PCR

We used the Plant RNA Kit (Huayueyang Biotechnology Co., LTD.) to extract the total RNA. Then, a total of 1.0 μg RNA was used for synthesizing cDNA according to the manufacturer’s protocol (PrimeScript RT regent Kit with gDNA Eraser (TaKaRa)). Primers were designed by online Primer-Blast software (https://www.ncbi.nlm.nih.gov/tools/primerblast/index.cgi?LINK_LOC=BlastHome) and produced by Genewiz Company (China). qRT-PCR was performed on the CFX96™ Real-Time System (Bio-Rad Laboratories) and the reaction conditions: 40 cycles of 95 °C for 5 s, 58 °C for 30 s, and 72 °C for 30 s. Each reaction mix contained 2.0 μL previously diluted cDNA (1:5), 7.5 mM of each primer, 12.5 μL TB Green Premix DimerEraser (Perfect Real Time; TaKaRa), and 9.0 μL RNase-free water to a final volume of 25 μL. At least three biological replicates for each gene was performed, all of the primers were listed in Table S5. We used *Actin* 18S rRNA as the reference genes [65]. SPSS 18.0 software was used for significance analysis. The 2^−ΔΔCt^ method was used to calculated the relative gene expression values [66].

### 4.7. Quantification of the JA, SA Contents, and Cellulose Content

The contents of JA and SA were measured according to the methods of You et al. [67], and the content of cellulose was measured according to the methods of Wei [68] at Cominbio Biotechnology Co., Ltd., Suzhou, China. Briefly for JA and SA, 0.1 g calli samples were weighed and ground in a mortar with 1 mL 90% methanol and then extracted overnight at 4 °C. Then, the sample was centrifuged at 8000× *g* for 5 min, the supernatant was taken out and the residue was leached with 0.5 mL 90% methanol for 2 h, and combined the twice supernatant. After decompression at 40 °C, the supernatant was evaporated to no organic phase (about 0.3 mL aqueous solution), adding 20 μL 1 mg/mL trichloroacetic acid aqueous for shocking 1 min. Then added 1 mL ethyl acetate and cyclohexane mixture (1:1 *v*/*v*) to extract twice, transferred the upper organic phase to the new EP tube, and dried with liquid nitrogen. A volume of 0.5 mL methanol (0.1% methane acid) was used to dissolve the dried pellets. Then, the product was detected by combined high-performance liquid chromatography (HPLC) after 0.22 micron filtration. HPLC analysis was carried out for the purified product with a 10 μL injection volume. The conditions were as follows: flow rate, 0.8 mL/min; column temperature, 35 °C; aliasing time, 30 min; wavelength of the ultraviolet detector was 230 nm for JA, and the fluorescence detector, excitation wavelength 294 nm, and emission wavelength 426 nm for SA.

For cellulose, 0.3 g calli samples were ground rapidly in a mortar with 1 mL 80% ethanol, then water bath at 95 °C for 20 min, and cooling to room temperature. The sample was centrifuged at 4000× *g* at 25 °C for 10 min, the supernatant was taken out and the residue was centrifuged again with 1.5 mL 80% ethanol and acetone, the precipitation was the coarse cell wall. Adding 1 mL acetic acid and soaking for 15 h to remove the starch, then the sample was centrifuged at 4000× *g* at 25 °C for 10 min, discarded the supernatant and dried the precipitation, and weighed the cell wall material. Then, 5 mg cell wall material was fully ground the homogenate with distilled water, fixed the homogenate to 0.5 mL with distilled water and placed in an ice bath. Slowly added 0.75 mL concentrated sulfuric acid, mixed well and let stand for 30 min in the ice bath, centrifuged at 8000× *g* at 4 °C for 10 min, took the supernatant and diluted it by 20 times with distilled water to be measured. Using ultraviolet spectrophotometer to detect the content and the wavelength of the ultraviolet detector was 620 nm.

## 5. Conclusions

We found that the expression of MdMLP423 was inhibited by BB and AAAP infection. Apple tissue overexpressing *MdMLP423* showed higher susceptibility to BB and AAAP than the CK and *MdMLP423*-overexpressing calli also exhibited weaker phytohormone signaling pathways and cell wall reinforcement. Additionally, we found that overexpressing *MdMLP423* inhibited the expression of a number of pathogenesis-related transcriptional factors and genes, which resulted in the calli being more sensitive to fungal infection. This study provides a foundation for the research of biotic stress-related gene resources in apples.

## Supplementary Materials

Supplementary materials can be found at https://www.mdpi.com/1422-0067/21/5/1879/s1.

## Abbreviations

MLP/RRP	Major latex protein/ripening-related proteins
Gly-rich-loop	Glycine-rich loop
BB	Botryosphaeria berengeriana
AAAP	Alternaria alternata apple pathotype
DEG	Differentially expressed gene
HPI	Hours post inoculation
SA	Salicylic acid
JA	Jasmonic acid
ABA	Abscisic acid
GA	Gibberellin
ETH	Ethylene
GD	Golden Delicious apple
HF	Hanfu apple
HY	Huayue apple
NJ	Neighbor-joining
Mw	Molecular weight
pI	Isoelectric point
iTRAQ	Isobaric Tags for Relative and Absolute Quantitation
LTR	Low-temperature responsive element
ME	Meristem expression
DSR	*Cis*-acting element involved in defense and stress responsiveness
HPLC	High-performance liquid chromatography
