# Supplementary material for: Major Latex Protein MdMLP423 Negatively Regulates Defense against Fungal Infections in Apple
Source: Int J Mol Sci. 2020 Mar 10;21(5):1879. doi: 10.3390/ijms21051879 (PMC7084931; doi:10.3390/ijms21051879)
Supplement: Supplementary file 1 [file ijms-21-01879-s001.zip › Supplementary materials/Supplementary Figure.docx]

**Supplementary** **Figure 1.** Alignment of the MdMLP423 protein with its orthologous genes in *Arabidopsis* AtMLP43.
